# Alexithymia and Autistic Traits: Associations With Social and Emotional Challenges Among College Students

**DOI:** 10.3389/fnins.2021.733775

**Published:** 2021-10-21

**Authors:** Potheini Vaiouli, Georgia Panayiotou

**Affiliations:** Department of Psychology, Center for Applied Neuroscience, University of Cyprus, Nicosia, Cyprus

**Keywords:** alexithymia, autism, TAS-20, emotion regulation, social skills

## Abstract

**Background:** Alexithymia is a multifaceted personality construct defined by marked difficulties in identifying and describing feelings and in externally oriented thinking. Given its intrinsic role in social-emotional processing, alexithymia is now recognized as a trans-diagnostic trait in a range of neurodevelopmental disorders, including autism. Research has pinpointed to the co-occurrence of autism with characteristics typical of alexithymic normative samples, such as social-communication difficulties and decreased emotion regulation abilities. Nonetheless, the role of individual facets of alexithymia in predicting challenges in social communication functioning is still understudied.

**Methods:** In total, 275 young adults completed the Toronto Alexithymia Scale, the Autism Spectrum Quotient (short form), the Interpersonal Competence Questionnaire, and the Difficulties in Emotion Regulation Scale self-reported questionnaires for assessing alexithymic and autistic traits, social-communication abilities, and emotion regulation difficulties. We used regression models to establish cross-sectional associations between autism, alexithymia, and social-emotional difficulties. Also, we ran a parallel mediation analysis to determine whether the relationship between autistic traits and emotion regulations challenges are mediated by Alexithymia facets.

**Results:** Analysis showed a significant positive association between autistic traits and alexithymic traits and between autistic traits and emotion regulation difficulties while, as expected, autistic traits were negatively correlated with social skills. A significant relationship was found among the participants’ levels of alexithymia and their interpersonal skills with two of three alexithymic subscales significantly contributing to the model. Similarly, a significant relationship was found among alexithymia subscales and emotion regulation difficulties with all three alexithymia subscales being statistically significant. Finally, analysis on two mediator models indicated a significant effect of autistic traits on social skills mediated by alexithymic traits as well as a significant indirect effect of autistic traits on emotion regulation difficulties mediated by alexithymic traits.

**Conclusion:** The results of this study provide evidence of the influence of different alexithymic facets on the relationship between autistic traits and social-emotional challenges in young adults. Longitudinal studies may explore further alexithymia and its associations with social-emotional difficulties in autism as well as the potential implications of these findings in intervention and treatment programs.

## Introduction

Alexithymia is a multifaceted personality construct with a dimensional nature that negatively impacts affective processing, dimensions of emotional regulation and the interpretation and recognition of emotional stimuli (both verbal and non-verbal). First described by Sifneos (1973), the alexithymia construct is broken down into several facets, the most widely recognized of which are difficulties in identifying feelings, difficulties in describing feelings, and poor external oriented thinking (Bagby et al., 1994). Elevated levels of alexithymia are implicated in social-emotional and mental health outcomes, including a range of neurodevelopmental conditions that affect social and emotional understanding (Berthoz et al., 2011; Bird and Cook, 2013). That is, individuals with high alexithymic traits are reported to experience significantly more interpersonal difficulties, difficulties describing their emotions, poor affect regulation, and an impaired ability to recognize bodily sensations compared to those individuals with low alexithymic traits (i.e., Spitzer et al., 2005; Vanheule et al., 2007; Bird and Cook, 2013).

Given its intrinsic role in social-emotional processing, alexithymia is now recognized as a trans-diagnostic risk factor in a range of mental health including autism. Autism spectrum disorders (ASD) are among the most common neurodevelopmental disorders with prevalence rates ∼1.9%, (Maenner et al., 2020) characterized by increased difficulties in the emotional and social domain [American Psychiatric Association (APA), 2013]. In addition, the term broader autism phenotype (BAP) is used to describe individuals who display clinical or personality characteristics similar to those typical of ASD, although it is not a formal diagnosis (Parr and Le Couteur, 2013). Individuals with BAP or autistic traits frequently present challenges in social communication and social relating, and have increased emotional difficulties, such as reduced emotion regulation abilities (Samson et al., 2012), along a spectrum of severity.

Elevated levels of alexithymia are a robust finding in adult populations with autism, with prevalence rates of alexithymia in the autism population being between 65 and 85% (Bird and Cook, 2013; Brewer et al., 2015; Kinnaird et al., 2019); higher prevalence among relatives of individuals with autism (Szatmari et al., 2008) compared to the general population. Several studies that examined autistic traits as a continuum rather than focusing on autism diagnosis, also found a strong association between alexithymic and autistic traits (see Shah et al., 2016; Nicholson et al., 2018). There are several pathways through which this association could be explained, for example, that alexithymia is the etiology of the socio-emotional difficulties associated with autistic traits, or vice versa, or that the link is indirect, and explained by other shared characteristics between autism and alexithymia. For example, alexithymia itself may cause anxiety and related sleep issues (i.e., Tani et al., 2004), and the difficulty to externalize and process emotions may lead to a variety of psychosomatic manifestations (Poquérusse et al., 2018). Further, emotion processing difficulties, which is a central alexithymic trait, are correlated with depression, which may ultimately be the common link that associates alexithymia and autism (Hill et al., 2004).

Suggesting a more direct path between the two traits, Bird and Cook (2013) have proposed that emotion processing deficits in autism stem from co-occurring alexithymia rather than ASD, known in the literature as the “alexithymia hypothesis.” Individuals with high alexithymic traits experience significantly more social-emotional difficulties than those with lower alexithymic traits (i.e., Spitzer et al., 2005), similar to individuals with high levels of autistic traits. Research findings indicate that alexithymic individuals may have a limited repertoire of emotion regulation skills (Darrow and Follette, 2014) involving inflexibility and disruptions in emotion processing (Panayiotou and Constantinou, 2017; Panayiotou et al., 2020) and poor emotion awareness (da Silva et al., 2017). Finally, studies that explored language in alexithymia have identified consistent challenges in language expression such as reduced complexity, openness and emotional content (Luminet et al., 2021), which are linked to deficits of empathy (Grynberg et al., 2018), core challenges found in autism as well.

Evidence in support of the alexithymia hypothesis, has begun to accumulate. Alexithymia total scores and scores on alexithymic facets appear to explain a number of difficulties in the processing, regulation and social expression of emotion, in individuals either meeting the autism diagnosis or characterized by autistic traits. Alexithymic traits have been shown to relate to difficulties in the production of emotion expressions (Trevisan et al., 2016) and empathy toward others (Bird et al., 2010) in individuals with autism. These skills are considered prerequisites for successful relationships and social interactions during daily routines (Trevisan et al., 2016) and their absence is known to disrupt everyday social functioning. Also, associations have been found between parent-reported alexithymic traits and the Autism Diagnostic Observation Schedule (ADOS) assessment scores (Hobson et al., 2020).

Looking at the role of alexithymia facets, the difficulty in identifying and describing feelings facet has been associated with difficulties in emotional reactivity and emotional regulation (Samson et al., 2012), difficulties in recognizing verbal and non-verbal emotional expressions (Heaton et al., 2012; Cook et al., 2013; Oakley et al., 2016), and challenges in the ability to experience and understand emotions (Herbert et al., 2011) in autistic individuals. However, more research is required to evaluate this hypothesis, especially with regards to the specific role of alexithymic facets, on particular difficulties associated with autistic traits, and with regards to individuals with different levels of autistic traits, when looking at them as a continuum of characteristics, found in the general population.

### The Current Study

In spite of the emergence of work on the association between facets of alexithymia and specific difficulties in autism (i.e., Liss et al., 2008; Oakley et al., 2020), more work is required to fully understand how sub-factors of these two constructs are related, as well as which are unrelated and distinct. In this study we examine alexithymic and autistic traits as a continuum of characteristics within the general population of young adults in Cyprus (college students), in order to provide data that will further clarify the nomological network of the link between the difficulties encompassed by the two constructs. Establishing that autism and alexithymia are indeed related but distinct allows us to make valid arguments in cases in which one construct can be used to explain difficulties related to the other. As both alexithymia and autism are conceptualized as being in a continuum, conducting such studies in the general population can help establish hypotheses on the contribution of alexithymia to the difficulties of autistic individuals that can then be tested in clinical samples.

Specifically, we aimed to assess cross-sectional associations between alexithymia (including its individual facets) and socioemotional and communication difficulties frequently observed in the BAP, including emotion regulation difficulties and interpersonal social skills, in a cohort of neurotypical young adults with varied levels of autistic and alexithymic characteristics. Extending previous literature on the co-occurrence of autistic and alexithymic traits, we focused on the followings: (1) Characterize the degree of association between alexithymic and autistic traits in the population of young, neurotypical colleges students of both genders. (2) Examine which facets of alexithymia are most strongly related to autistic traits and to difficulties typically related to autism. (3) Examine which facets of alexithymia may help explain (i.e., mediate) the association between autistic traits and emotional/interpersonal difficulties, specifically emotion regulation and social skills.

## Materials and Methods

### Participants

A total of 275 Greek-Cypriot undergraduate university students (73.8% females; *M*age = 21.01, SD = 2.49 years) were recruited from introductory undergraduate courses of psychology and received extra course credit or were included in a prize-draw for their participation. Students pass competitive exams to enter the particular university, while those who enter based on disability criteria meet a specific performance cutoff relative to their cohort; therefore, although the intellectual ability of participants was not assessed, all students can be assumed not to have significant diversions from at least average intellectual ability. The majority of them were women (see Table 1), which is consistent with the composition of the student body at the particular university. From the total pool, 23 cases (9.1%) were excluded from analyses due to incomplete questionnaires on the autism measure. In total, 252 participants with available data on all measures were included in this study. No further exclusion criterial were involved and both autistic traits and alexithymia were assessed *via* self-report, conceptualized as continuous traits. Therefore, no clinical diagnostic procedures were followed. The study is part of a larger project on personality and mental health that received approval from the Cyprus National Bioethics Committee. Table 1 shows the sample’s characteristics.

**TABLE 1:** Correlations among all variables included in the study.

		1	2	3	4	5	6	7	8	9	10	11	12	13	14	15	16	17	18
1	Autism																		
2	Alexithymia	0.206**																	
3	TASDIF	0.202**	0.846**																
4	TASDDF	0.193**	0.847**	0.617**															
5	TASEOT	0.045	0.522**	0.123*	0.250**														
6	DESRS total	0.185**	0.669**	0.698**	0.523**	0.205**													
7	DERS non-acceptance	0.179**	0.430**	0.465**	0.353**	0.085	0.768**												
8	DERS goals	0.091	0.303**	0.350**	0.307**	–0.045	0.698**	0.448**											
9	DERS impulse	0.159*	0.539**	0.616**	0.363**	0.153*	0.856**	0.579**	0.525**										
10	DERS awareness	–0.008	0.445**	0.321**	0.303**	0.410**	0.406**	0.079	0.086	0.252**									
11	DERS strategies	0.200**	0.529**	0.576**	0.419**	0.116	0.888**	0.686**	0.627**	0.748**	0.152*								
12	DERS clarity	0.160*	0.768**	0.778**	0.608**	0.261**	0.772**	0.497**	0.344**	0.613**	0.423**	0.600**							
13	ICQtotal	−0.204**	−0.383**	−0.223**	−0.354**	−0.324**	−0.340**	−0.238**	–0.117	−0.219**	−0.365**	−0.286**	−0.324**						
14	ICQ initiation	−0195**	−0.320**	−0.215**	−0.322**	−0.195**	−0.248**	−0.144*	–0.090	−0.129*	−0.269**	−0.215**	−0.294**	0.854**					
15	ICQ.NegAssert, Interpersonal Competence Questionnaire, Negative Assertion.	−0.183**	−0.315**	−0.151*	−0.211**	−0.415**	−0.289**	−0.245**	–0.026	−0.205**	−0.336**	−0.231**	−0.277**	0.821**	0.573**				
16	ICQ disclosure	−0.177**	−0.282**	−0.184**	−0.228**	−0.249**	−0.315**	−0.240**	−0.135*	−0.196**	−0.329**	−0.268**	−0.257**	0.867**	0.739**	0.664**			
17	ICQ emotional support	–0.109	−0.304**	–0.090	−0.399**	−0.249**	−0.137*	–0.112	–0.017	–0.018	−0.231**	–0.118	−0.152*	0.746**	0.585**	0.466**	0.519**		
18	ICQ conflict management	−0.168**	−0.342**	−0.284**	−0.272**	−0.214**	−0.422**	−0.244**	−0.227**	−0.376**	−0.331**	−0.357**	−0.356**	0.793**	0.568**	0.660**	0.627**	0.427**	

**Correlation is significant at the 0.01 level (two-tailed); *Correlation is significant at the 0.05 level (two-tailed).

### Measures

#### Alexithymia

The 20-item self-report Toronto Alexithymia Scale (TAS-20; Bagby et al., 1994) was administered to measure alexithymia. The TAS-20, the most frequently used measure of alexithymia in adults and in prior research pertaining to autism (Vaiouli and Panagiotou, 2021) results in a total score and three subscales: difficulties in identifying feelings, difficulties in describing feelings, and externally oriented thinking. Higher scores indicate higher alexithymia, with scores ≥ 61 indicating “severe” (i.e., clinically relevant) alexithymia (Parker et al., 2003).

#### Autism Traits

The short form of the Autism Spectrum Quotient questionnaire (Baron-Cohen et al., 2001) for adults (AQ-10) was used for measuring the severity of autistic traits. The self-report questionnaire assesses the potential neurodevelopmental impairment with 10 items rated on a 4-point-Likert scale ranging from 1 (I strongly disagree) to 4 (I strongly agree). Total scores range from 0 to 5, such that lower scores on AQ indicate fewer autistic traits; total scores above 6 indicate more autistic traits. For this study, the total score was used as a continuous measure of symptom severity. The scale had very good internal consistency with a Cronbach’s α = 0.90 for the current sample. The AQ-10 was used instead of the original Autism Spectrum Quotient (Baron-Cohen et al., 2001) as the AQ-10 was more economic with only 10 items and there is little performance difference between the AQ-10 and the full AQ-50 (Booth et al., 2013).

#### Emotion Regulation Difficulties

The Difficulties in Emotion Regulation Scale (DERS; Gratz and Roemer, 2004) is a 36-item, self-report measure. It was utilized to assess participants’ emotion regulation deficits. The DERS provides an overall emotion dysregulation score, and scores for six factors that reflect the multifaceted nature of emotion regulation: non-acceptance of emotional responses (Non-acceptance); difficulties engaging in goal-directed behavior whilst distressed (Goals); impulse control difficulties whilst distressed (Impulse); lack of emotional awareness (Awareness); limited access to emotion regulation strategies (Strategies); and lack of emotional clarity (Clarity). For our sample, reliability was excellent 0.93.

#### Social Skills

This 40-items Interpersonal Competence Questionnaire (ICQ) scale assessed participants’ social skills (Buhrmester et al., 1988), through a 5-point scale (1 = I am poor at this, 5 = I am extremely good at this). ICQ involves five subscales; initiating relationships, providing emotional support, asserting influence, self-disclosure, and conflict resolution (eight items per subscale). In the standardization study, internal consistency for the five domains of the ICQ was good, ranging from 0.77 to 0.87 (Buhrmester et al., 1988). For our sample, reliabilities for the five subscales were good to excellent ranging between 0.89 and 0.94.

### Procedure

The study was completed in one phase of questionnaire completion over a period of 3 months. Participants were invited to complete the aforementioned self-report questionnaires, as part of a larger study. All questionnaires were administered to the participants in the same order through an online platform (LimeSurvey) and the duration for the full package was approximately 2 h. Informed consent was obtained by all participants.

### Plan of Analyses

Data processing and analysis was performed using SPSS v25 and RStudio (statistical programming language R). First, we performed Pearson’s *r* bivariate correlations among study variables to examine associations among the study constructs. Then, we addressed the respective associations between autistic and alexithymic traits and socioemotional difficulties, using multiple linear regression models. Dependent variables were in two separate models, respectively, social skills and emotion regulation difficulties, i.e., the negative socio-emotional outcomes often associated with autism spectrum. Independent variables were age, gender, alexithymia, and autism trait scores.

**FIGURE 1:**
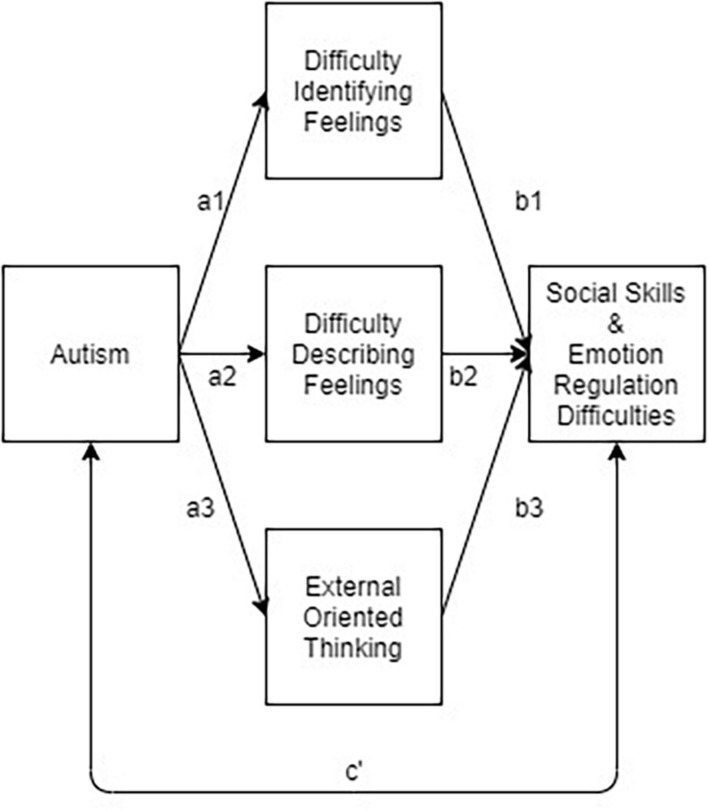
Proposed mediation model of the relationship between ASD traits (AQ), alexithymic traits (TAS-20) and social skills (ICQ), and emotion regulation abilities (DERS). Separate analyses were conducted for the ICQ and DERS, respectively.

Finally, to determine whether the relationship between autistic traits and social-emotional difficulties were mediated by alexithymic traits (TAS-20 DIF, DDF, and EOT), two structural equation modeling (SEM) models were conducted with R studio. In each of the two models, the three TAS-20 subscales were entered as mediators between AQ and ICQ (first model) or DERS (second model) (Figure 1). Given the cross-sectional nature of the study, our ordering of the variables in the model was based on both theoretical and developmental considerations. As per the “alexithymia hypothesis” alexithymia is believed to explain (i.e., is the mechanism) the link between autism and negative socio-emotional outcomes, therefore indicating alexithymia as the mediator. Furthermore, autistic traits are considered largely hereditary (Colvert et al., 2015), and its effects on development can be seen early in life. Although less is known about the developmental origin of alexithymia, several models suggest that it may be a result of early traumatic experiences, or an acquired coping approach involving pervasive avoidance of emotions (Badura, 2003; Krystal, 2015; Panayiotou et al., 2020), therefore it is assumed to appear somewhat later in life than autism characteristics.

## Results

### Associations on Social-Emotional Challenges, Alexithymic, and Autistic Traits

Pearson’s *r* correlation examined associations among all variables in the study and showed a significant positive association between autistic traits and alexithymic traits (*r* = 0.21, *p* < 0.01), which however is not high enough to suggest multicollinearity; this finding addressed question 1, but showing that the two constructs are related but independent. There were also significant associations between autistic traits and emotion regulation difficulties (DERS) (*r* = 0.18, *p* < 0.05) while, as expected, autistic traits were negatively correlated with social skills (ICQ) (*r* = −0.20, *p* < 0.01).

Specifically, there were significant negative associations between autistic traits and all facets of social skills, including the ability to initiate and sustain social interactions and manage conflicts (as measured by subscales of the ICQ). Also, autistic traits positively and significantly correlated with two of the three facets of alexithymia (i.e., Difficulty Identifying Feelings and Difficulty Describing Feelings), showing once more the relatedness between the two traits; in response to question 2, and in accord with the few previous studies that addressed this issue, results concur that among neurotypical young adults, the strongest association is between autistic traits and the two “emotional” facets of alexithymia. Furthermore, autistic traits were significantly related to four aspects of emotion regulation difficulties (but not with the difficulty in awareness and goals; see Table 1).

#### Prediction of Social Skills/Emotional Difficulties by Alexithymic and Autistic Traits

To examine the role of alexithymia and its facets in the socioemotional difficulties associated with autism (question 3), we first conducted regression analyses to affirm that both autistic and alexithymic traits predict these forms of difficulties. Multiple-linear regressions were calculated to predict interpersonal social skills and emotion regulation challenges respectively, based on the self-reported levels of alexithymia and autism of the participants. Gender and age were entered as covariables but they were not statistically significant in any of the models and they were completely removed from the models. Also, exploratory analyses (i.e., repetition of the regression analyses described here) were run separately for each gender, and similar associations were found to hold for both genders. Therefore, gender is not further discussed here, and our unequal gender samples do not appear to have affected outcomes of main analyses.

In model 1, alexithymic subscales were entered as the predictors and interpersonal social skills were the outcome. A significant relationship was found among the participants’ levels of alexithymia and their interpersonal skills [*F*(3,251) = 19.17, *p* < 0.01], *r*^2^ = 0.17 with two of three alexithymic subscales significantly contributing to the model (DDF: *B* = −1.56, *p* < 0.01; EOT: *B* = −1.65, *p* < 0.01) while Difficulty in Identifying Feelings did not (*B* = −0.008, *p* = 0.98).

In model 2, we ran the same model but entered all alexithymic subscales as predictors of the emotion regulation challenges (as measured by DERS). Similarly, a significant relationship was found among alexithymia subscales and emotion regulation difficulties [*F*(3,270) = 92.08, *p* < 0.01], *r*^2^ = 0.51. All three subscales of alexithymia were statistically significant predictors (DIF: *B* = 2.23, *p* < 0.01; DDF: *B* = 0.55, *p* < 0.05, and EOT: *B* = 0.59, *p* < 0.05).

Next, we ran two additional models in which autistic traits (total scores) were entered as the predictors with interpersonal social skills and emotion regulation challenges respectively as the outcomes. A significant relationship was found among the participants’ levels of autism and their interpersonal skills [Model 3: *F*(1,251) = 10.81, *p* < 0.01], *r*^2^ = 0.04 as well as the participants emotion regulation challenges [Model 4: *F*(1,251) = 8.81, *p* < 0.01], *r*^2^ = 0.03. Table 2 presents all information on the regression analysis.

**TABLE 2:** Regression coefficient for autistic and alexithymic traits, as predictors of social and emotional skills.

		*B*	SE	Beta	*p*<
**Model 1**	TAS DIF	–0.008	0.31	–0.002	0.98
	TAS DDF	–1.56	0.39	–0.29	0.00
	TAS EOT	–1.65	0.38	–0.25	0.00
	*r*^2^ = 0.177				

**Model 2**	TAS DIF	2.23	0.20	0.61	0.00
	TAS DDF	0.55	0.25	0.12	0.03
	TAS EOT	53	0.24	0.09	0.03
	*r*^2^ = 0.503				

**Model 3**	Autism	–3.55	1.08	–0.20	0.00
	*r*^2^ = 0.038				

**Model 4**	Autism	2.73	0.92	0.18	0.00
	*r*^2^ = 0.030				

### Structural Equation Modeling

Following the verification of the presence of significant associations between both the predictor (autistic traits) and the proposed mediator (alexithymia) with the outcome variables by the regression analyses above, SEM was used to determine whether the relationship between autistic traits (as measured by the AQ) and social skills (ICQ) and emotion regulations challenges (DERS), respectively are significantly mediated by Alexithymia factors (TAS-20, DIE, DDE, and EOT), and which ones. We ran two different SEM models, using a bootstrap resampling value of 10,000, and 95% bias-corrected confidence intervals (CIs).

**FIGURE 2:**
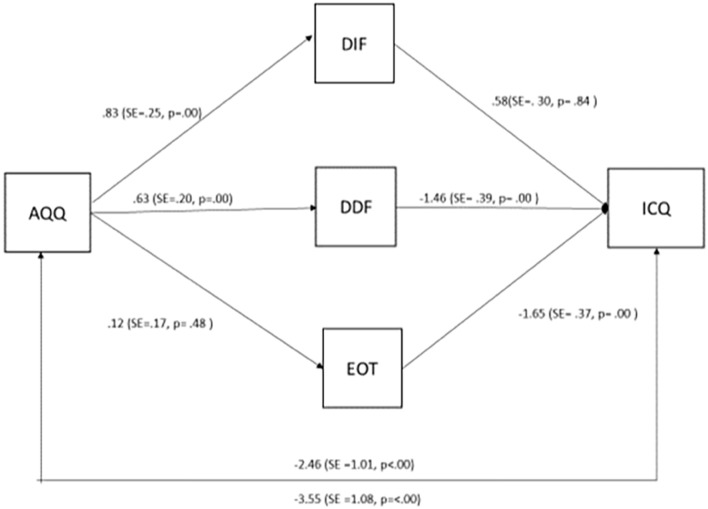
Structural equation model 1.

In the first mediator model (Figure 2), autistic traits were the predictors and social skills were the outcome, mediated by the three alexithymia subscales. The analysis verified that autistic traits were a significant independent predictor on social skills (*B* = −2.46, SE = 1.01, *p* < 0.005). As predicted, there was also a significant indirect effect of autistic traits on social skills mediated by alexithymic traits (*B* = −3.55, SE = 1.08; *p* < 0.005) with Difficulty Describing Feelings being a significant [CI (−1.92, −0.21)] mediator, while Difficulty Identifying Feelings [CI (−0.49, 0.63)] and Externally Oriented Thinking [CI (−0.76, 0.33)] not being significant.

**FIGURE 3:**
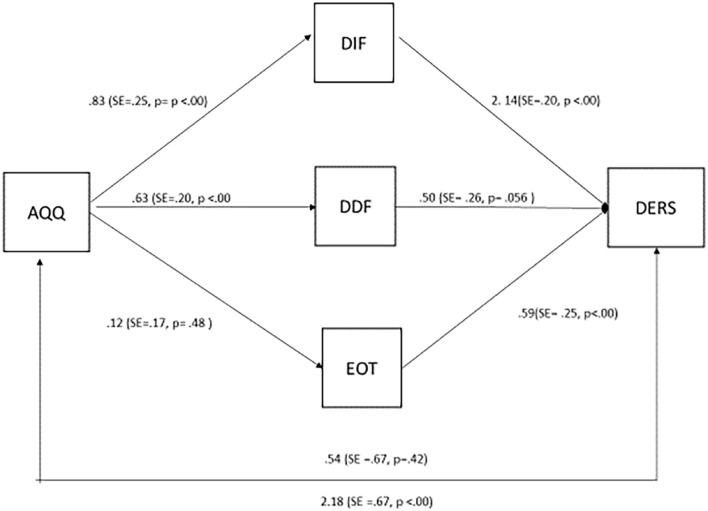
Structural equation model 2.

In the second mediator model (Figure 3) autistic traits were entered as the predictors of emotion regulation difficulties, mediated by alexithymic traits. Autistic traits were not a significant direct predictor on Emotion Regulation Difficulties (*B* = 0.54, SE = 0.67, *p* = 0.42). There was a significant indirect effect of Autistic traits on Emotion Regulation Difficulties mediated by Alexithymic traits (*B* = 2.18, SE = 0.67; *p* ≤ 0.05) with DIF being a significant mediator [CI (0.72, 2.99)], while DDF [CI (−0.43, 0.83)] and EOT [CI (−0.12, 0.32)] were not. The latter model indicates that the association between autistic traits and emotion regulation difficulties is not direct but fully mediated by the presence of specific alexithymic characteristics.

## Discussion

### Alexithymia and Social-Emotional Difficulties in Autism

This study extends existing work that examines the role of alexithymia in the relationships between autism and socio-emotional difficulties, specifically, emotion regulation and social skills. In this study, we conceptualized autistic traits on a continuum of severity, found in neurotypical young adults, and therefore aimed to show that similar association among alexithymia and autistic difficulties can be found not only among diagnosed individuals with autism, but at all levels of severity of the autism spectrum. The results provide specific evidence of the influence of different alexithymic facets on the relationship between autistic traits and social-emotional challenges in the young adults in our sample. To our best knowledge this is one of the few studies to date to assess cross-sectional associations between social-emotional difficulties and all alexithymic facets, beyond the total score in alexithymia scales (see also Oakley et al., 2020). While other studies have provided similar results (i.e., Liss et al., 2008; Kinnaird et al., 2019), suggesting that alexithymia is an important trait with implications for individuals on the autism spectrum, we identify the importance of specific facets of alexithymia in relation to specific social and emotional difficulties.

First, we found that, as expected based on prior research, autistic traits and alexithymia aspects are indeed significantly associated, but not multicollinear (associations <0.8, typically considered as the criterion for multicollinearity), suggesting related but independent constructs. Next, we found that autistic traits and the participants’ self-reported social and emotional difficulties were not only associated with the total alexithymia scores but most strongly with two of the three facets of alexithymia (that is difficulties in identifying/describing feelings). Research that looked specifically into facets of alexithymia in autistic individuals have provided similar results, indicating that the “emotional” aspects of alexithymia, difficulties with describing and identifying feelings, are also the facets most strongly associated with social and emotional challenges (i.e., Kloosterman et al., 2009; Milosavljevic et al., 2016; Schaller and Rauh, 2017).

These findings provide evidence on the intersection between alexithymic traits and common characteristics of autism, especially in the social and emotional domain. They indicate that alexithymia is closely related to the emotional/social challenges involved in autism while lack of significant evidence on the externally oriented thinking facet weakens the associations between alexithymia and cognitive challenges of individuals with autistic characteristics. For example, autistic individuals experience challenges in recognizing specific emotions, such as fear and disgust (Humphreys et al., 2007; Wallace et al., 2008), anger (Ashwin et al., 2006), or sadness (Corden et al., 2008), in interpreting facial expressions, and in describing the emotional content of verbal, auditory, and visual stimuli (Allen et al., 2013; Cook et al., 2013). Notably those are pre-requisites for successful emotional and social understanding. As such, one possible explanation for the observed associations between alexithymia and social-emotional difficulties is that hindered communication, challenges in social relationships, and disruption of everyday social interactions (Trevisan et al., 2016) which have been associated with autistic traits, may actually be interpreted by co-occurring emotional facets of alexithymia.

Of interest to this study, different facets of alexithymia were related with different aspects of social and emotional challenges in our findings. Specifically, two alexithymic subscales (difficulty describing feelings and externally oriented thinking) were found to predict participants’ interpersonal social skills and all alexithymic subscales were found to predict participants’ emotional regulation challenges. Social interactions are complex phenomena, requiring both emotional and cognitive skills (e.g., ability to empathize, and ability to pay attention to conversation). It appears that both the emotional and cognitive facets of alexithymia, i.e., the difficulty in verbally communicating emotion, and the difficulty in being attentive to ones’ internal experiences (in order to know them and communicate them), disrupt the ability to socially interact. In the case of skills to regulate one’s own emotions, all aspects of alexithymia had negative effects, as emotion regulation implicates a wide range of strategies (as measured here by the DERS) that may rely on different sorts of abilities (e.g., difficulty describing feelings may hinder emotional sharing, communication and support seeking, difficulty in identifying feelings may hinder emotional awareness and acceptance, and externally oriented thinking, may be associated with avoidance of internal experiences, leading to their poor regulation).

However, only the difficulty in describing feelings facet significantly mediated the relationship between autistic traits and social skills and only the difficulty in identifying feelings facet significantly mediated the relationship between autistic traits and emotion regulation difficulties in our sample. These effects stress the role of the ability to communicate emotions, in the interpersonal difficulties of individuals with autistic traits, and the ability to “know” one’s emotions, for effective emotion regulation (Barrett, 2004). In both cases we observed significant mediation by alexithymia facets, in support of the alexithymia hypothesis, which argues that it is alexithymia that may be responsible for the socio-emotional challenges in autism. Importantly, in model one, both direct and indirect associations were found between autistic traits and social skills– suggesting that the alexithymia hypothesis is supported partially: that is, autistic traits themselves contribute directly to these difficulties over and above the indirect association *via* alexithymia characteristics. In the case of emotion regulation difficulties, however, the direct path between autistic traits and these difficulties was not significant, showing that these difficulties may be fully described by alexithymia.

The co-occurrence of autism with characteristics typical of alexithymia, such as difficulty identifying emotions (Silani et al., 2008; Kloosterman et al., 2009), difficulties with emotion regulation (Weiss et al., 2014; Costa et al., 2017), and decreased levels of empathy (Lartseva et al., 2015) is well-documented in the literature. Collectively, these findings align with the conceptualization of alexithymia as pertaining to emotional difficulties and a growing body of research that explores the alexithymia hypothesis (Bird and Cook, 2013), according to which the emotion processing deficits in autism stem from co-occurring alexithymia rather than autism.

Findings like our own, also suggest possible directions for potential interventions, for individuals with various degrees of autistic traits. As suggested by our mediation models, training in the ability to recognize and describe emotions, perhaps through psycho-educational activities, and in staying attuned to one’s internal experiences, in order to know, process, and ultimately accept them (e.g., through meditation), may be of paramount importance in addressing the interpersonal and emotional challenges encountered in individuals with autistic traits. It remains to be seen, if our models can replicate at higher levels of clinical autistic characteristics, which would suggest similar interventions for autism spectrum.

## Conclusion

Over the last decades, special attention has been given to the complex emotional abilities of autistic individuals, providing evidence for deficits in the basic processing of emotions, abnormalities in emotional reactivity and emotional regulation (i.e., Samson et al., 2012). However, the complexity and intensity of deficits do not appear to be universal within the autistic population. In line with the alexithymia hypothesis (Bird and Cook, 2013), the strongest explanation for this inconsistency is alexithymia. It may be necessary for future studies on this and related areas to control for alexithymia and particularly its three facets in their design and analysis. That will allow to explore the extent to which the manipulation of specific aspects of emotional processing could improve emotional responding with a specific focus on clinical applications. This includes recognizing alexithymic traits in the clinical and therapeutic care of individuals with autistic traits, as alexithymia may act as a modifier, negatively affecting social and emotional difficulties in autism (Costa et al., 2017; Oakley et al., 2020).

### Limitations and Future Steps

The current study had several limitations. Participants were not explicitly asked to report whether they had diagnosis of ASD. While high scores on AQ may indicate more autistic traits, autistic individuals may report low on AQ scores, or have received social skills interventions and therefore report exhibiting fewer traits. Further, this study included a non-clinical sample and it is not clear whether the relations reported in this largely non-autistic sample would hold for a clinical sample. In addition, this was a relatively homogeneous sample (consisting of a larger proportion of women), and it is unclear whether the relationships observed would be found in a more diverse group. Also, this study was cross-sectional; therefore, no inferences about the causal relationships between the variables investigated can be made.

Regarding measurement, emotion identification is considered a counter-intuitive approach for measuring constructs associated with problems on emotion identification (Griffin et al., 2016). This may introduce further bias than what is inherent in self-report measures, as participants may have challenges to assess their own emotional abilities, either due to alexithymic and autistic traits or other variables (e.g., self-esteem and over or under-reporting of their perceived qualities). Further, work for adolescents and children with autism has indicated that parent and child reports of alexithymia do not correlate (Griffin et al., 2016; Hobson et al., 2020; Hobson and Bedem, 2021). This highlights further the issue of self-reporting these variables, especially in clinical samples and pinpoint to the need for multiple data sources and the further development of behavioral measures to assess the alexithymia construct in younger ages.

Overall, the results are preliminary and need to be verified in longitudinal research. Investigating how alexithymic traits interact with dimensions of affective processing, physiology, and emotion regulation through a multi-method approach provides important directions in research and insights on social-emotional challenges of autistic individuals with a specific focus on clinical applications. Future research may focus on the associations between the two traits using both a longitudinal and rigorous experimental approach with the inclusion of clinical autism. This would help understand both autism and alexithymia and why they overlap, clarify how much of deficits alexithymia may explain in autism, and explore social and emotional challenges in autistic individuals with the potential to provide novel directions in clinical interventions and research.

## Data Availability Statement

The raw data supporting the conclusions of this article will be made available by the authors, without undue reservation.

## Ethics Statement

The studies involving human participants were reviewed and approved by the Cyprus National Bioethics Committee. The patients/participants provided their written informed consent to participate in this study.

## Author Contributions

Both authors listed have made a substantial, direct and intellectual contribution to the work, and approved it for publication.

## Conflict of Interest

The authors declare that the research was conducted in the absence of any commercial or financial relationships that could be construed as a potential conflict of interest.

## Publisher’s Note

All claims expressed in this article are solely those of the authors and do not necessarily represent those of their affiliated organizations, or those of the publisher, the editors and the reviewers. Any product that may be evaluated in this article, or claim that may be made by its manufacturer, is not guaranteed or endorsed by the publisher.
